# Understanding evidence: a statewide survey to explore evidence-informed public health decision-making in a local government setting

**DOI:** 10.1186/s13012-014-0188-7

**Published:** 2014-12-14

**Authors:** Rebecca Armstrong, Elizabeth Waters, Laurence Moore, Maureen Dobbins, Tahna Pettman, Cate Burns, Boyd Swinburn, Laurie Anderson, Mark Petticrew

**Affiliations:** Jack Brockhoff Child Health and Wellbeing Program, Melbourne School of Population and Global Health, University of Melbourne, Melbourne, VIC Australia; MRC Unit for Social and Public Health Sciences, University of Glasgow, Glasgow, Scotland; School of Nursing, McMaster University, Hamilton, ON Canada; School of Dentistry and Health Services, Charles Sturt University, Wagga Wagga, New South Wales Australia; Population Nutrition and Global Health, University of Auckland, Auckland, Grafton New Zealand; New Zealand and WHO Collaborating Centre for Obesity Prevention, Deakin University, Geelong, VIC Australia; Department of Epidemiology, School of Public Health, University of Washington, Seattle, WA USA; London School of Hygiene and Tropical Medicine, University of London, Bloomsbury, London, UK

## Abstract

**Background:**

The value placed on types of evidence within decision-making contexts is highly dependent on individuals, the organizations in which the work and the systems and sectors they operate in. Decision-making processes too are highly contextual. Understanding the values placed on evidence and processes guiding decision-making is crucial to designing strategies to support evidence-informed decision-making (EIDM). This paper describes how evidence is used to inform local government (LG) public health decisions.

**Methods:**

The study used mixed methods including a cross-sectional survey and interviews. The Evidence-Informed Decision-Making Tool (EvIDenT) survey was designed to assess three key domains likely to impact on EIDM: access, confidence, and organizational culture. Other elements included the usefulness and influence of sources of evidence (people/groups and resources), skills and barriers, and facilitators to EIDM. Forty-five LGs from Victoria, Australia agreed to participate in the survey and up to four people from each organization were invited to complete the survey (*n* = 175). To further explore definitions of evidence and generate experiential data on EIDM practice, key informant interviews were conducted with a range of LG employees working in areas relevant to public health.

**Results:**

In total, 135 responses were received (75% response rate) and 13 interviews were conducted. Analysis revealed varying levels of access, confidence and organizational culture to support EIDM. Significant relationships were found between domains: confidence, culture and access to research evidence. Some forms of evidence (e.g. community views) appeared to be used more commonly and at the expense of others (e.g. research evidence). Overall, a mixture of evidence (but more internal than external evidence) was *influential* in public health decision-making in councils. By comparison, a mixture of evidence (but more external than internal evidence) was deemed to be *useful* in public health decision-making.

**Conclusions:**

This study makes an important contribution to understanding how evidence is used within the public health LG context.

**Trial registration:**

ACTRN12609000953235.

**Electronic supplementary material:**

The online version of this article (doi:10.1186/s13012-014-0188-7) contains supplementary material, which is available to authorized users.

## Background

As a concept, evidence-informed decision-making (EIDM) refers to the process of combining a range of sources of evidence to inform a decision [1-3]. In practice, this occurs within a political context that requires consideration of a range of other factors including research evidence, community views, budget constraints, and expert opinion [4-7]. Public health practitioners are increasingly encouraged to practice EIDM. In recent years, there has been a proliferation of literature including frameworks that describe EIDM processes and a number of systematic reviews to identify effective interventions [5,8-10]. However, in public health, there is limited understanding of the effects of these strategies in terms of increasing the contribution of research evidence to decision-making [5,9]. Whilst there has been investment in resources to support decision-makers such as online repositories and evidence summaries, the effort has not been delivered systematically. There are limited systems or infrastructure available to the public health workforce in Australia to support EIDM.

Three tiers of government operate in Australia: Commonwealth, State and Local. Local governments (LGs) operate locally meaning government of a town, city or region involving locally-elected officials. LGs are responsible for various local functions including planning and building approval (e.g. zoning of land), roads and parking, recreation and culture (e.g. swimming pools and public festivals), community services (e.g. maternal and child health), waste management and local laws. As such, LGs are similar to provincial public health departments in Canada and local authorities in the UK. Individuals working in LG public health teams come from very varied educational and professional backgrounds such as environmental science, sport and recreation, social planning, in addition to health promotion and public health specialists. This differs significantly from other jurisdictions dominated by medically trained public health practitioners (Canada and UK).

The objectives of this study were to identify the types of evidence used within LGs and to explore their relative contribution to the process of EIDM. The information garnered contributes to global knowledge about EIDM and informed the design of an exploratory cluster RCT (Knowledge Translation for LG—KT4LG) to be implemented in Victorian LG (Australia, New Zealand, Clinical Trials Register ACTRN12609000953235).

## Methods

### Study design

In order to explore the diverse research questions scientifically, a mixed-method design was applied; these are characterised by a series of projects complete in themselves but related to an overall project aim [11]. Data are collected concurrently, analysed separately, and results are compared during interpretation [12]. The purpose of the study is triangulation. The quantitative data was used to provide an overall picture of EIDM in LG and qualitative data was needed to corroborate quantitative findings and provide more in-depth understanding of the underpinning processes. Outcomes from the two data sets are then synthesized into final overarching findings [13].

### Theoretical frameworks

The overall theoretical approach for this study was informed by the Evidence-Informed Policy and Practice Pathway (EIPPP) [1] which was used to guide the exploration of policy influences, context and decision-making factors, and their impact on sourcing, using and considering capacity to implement within an evidence-informed framework [1].

Diffusion of innovations theory was used to help understand how EIDM might spread within these stages of the policy process and so informed Evidence-Informed Decision-Making Tool (EvIDenT) survey development and interview question design. It is increasingly used to help explore how ‘innovations’, which could be (depending on the perspective) research ideas or policy ideas, spread amongst individuals and organizations [1,14,15]. Modern interpretations acknowledge the non-linearity and complexity of ‘research into practice’ processes [16,17]. Diffusion theory is useful in helping to identify how influential/useful evidence might be in the decision-making process. In doing so, it is important to identify points at which knowledge translation interventions could be introduced to increase research use. Other theoretical frameworks are necessary to show the relationship between research and policy, including those that link policy and research utilization [1,18,19], evidence about EIDM practice [18,20] and models depicting processes of knowledge translation [21-23]. Together, these theoretical frameworks influenced the development of key domains: access, confidence and culture, the design of the questions, and interpretation of the results.

### Survey development

Informed by previous work [24,25], EvIDenT was designed to collect data about evidence use and decision-making processes in the LG context. It was based on three core domains representing key factors in individual and organizational decision-making: access to evidence, confidence in using evidence and organizational culture for using research evidence to inform decision-making. Additional areas of interest including skills, influence and usefulness of various sources of evidence and barriers and facilitators to EIDM were also included. Items were then developed to explore each of these dimensions (see Additional file 1). Likert scales were used to measure perceptions (level of agreement from 1 = ‘strongly disagree’ to 7 = ‘strongly agree’). The survey also included a demographics and work history. Methods for survey development and psychometric testing have been described elsewhere [26]. Open-ended questions were included to explore strategies that were perceived to facilitate EIDM and to identify additional strategies that could be employed. These informed the development of the KT4LG intervention [27]. Copies of the survey are available from the corresponding author.

### Sampling

LG was chosen as the setting for this study having an increasing emphasis on preventive health [28]. Given that evidence-use for decision-making was likely to vary by organizational types, this study would contribute to understanding EIDM processes operating at this level of government. Further, LGs work across sectors and settings and are required to apply a broad range of evidence across a large spectrum of issues relevant to their constituents. It was anticipated that understanding evidence use and influence in LG would provide some insight into the application of EIDM in multi-sector settings.

All 79 LGs across the state of Victoria, Australia, were invited to participate in the EvIDenT survey. Chief executive officers (CEOs) were mailed an information kit, outlining the intent of the project, the requirements of participation, a plain language statement about the study and an organizational consent form. Participating councils were asked to nominate up to four employees who were involved in *public health planning*, *policy or programs* and *who represented diverse work areas*. Follow-up phone calls were necessary to confirm participation and to ensure organizational consent and nomination forms were completed. Nominated employees within the LGs were emailed the plain language statement and a link to the online survey, which had an individual consent form built-in.

The sample for the interviews was drawn from survey participants who had nominated their interest in interview participation. During survey completion, all individual participants were asked to indicate their willingness to participate in an in-depth interview. Potential participants were invited by email within 2 weeks of survey completion.

Ethical approval was granted by the University of Melbourne Human Ethics Sub Committee [722362].

### Data collection from survey

Survey data were entered online by participants directly between November 2008 and April 2009 [29]. After the survey implementation period, data were exported to MS Excel, cleaned and then exported to Stata 10.1 for analysis [30]. Organizational characteristics of each LG were obtained from a centralized source [31] including data on population size, recurrent income, geographic size and location (metropolitan or rural areas).

### Data collection from interviews

Interviews were conducted by phone (by RA) and lasted between 45–70 min. Interviews focused on the implementation of EIDM, including defining evidence, and practices and processes for evidence-informed public health. Probes were used to stimulate discussion and clarify previous responses, for example: How is evidence defined by you? Does this differ from how it is defined by your organization? Although there was a theoretical structure for the content of the interviews, they did aim to establish a sense of reciprocity with interviewees in order to uncover and construct meaning of EIDM in this context. All interviews were digitally recorded and professionally transcribed.

### Analysis

Aligned with a concurrent triangulated research design, the overarching data analysis framework was parallel mixed analysis [32]. The quantitative and qualitative data were analysed separately, key themes from each data set were extracted and displayed (data reduction and display), quantitative data was transformed into key themes or a narrative (data transformation), findings for each data set were then compared to note differences and similarities (data comparison) and finally, the findings from the two data sets integrated (data integration) and related back to research questions and theoretical framework [33].

Quantitative data were analysed using Stata 10.1 [30]. Descriptive statistics including frequencies, proportions, means, and standard deviations were used to describe the characteristics of the individuals (participants) and organizations (LGs) and responses. Histograms were used to represent the distribution of responses to Likert scale questions measuring core domains: access, confidence, skills and organizational culture. Responses to usefulness, influences and barriers were tabulated, and mean scores were calculated.

All analyses were adjusted for clustering effects due to nesting that may have been caused by individual respondents being located within organizations (LGs). Intra-cluster correlation coefficients were also used to examine the correlation of individual responses from the same organizations [34] to identify whether individuals within councils had similar experiences or views about their organization’s culture for research evidence use in decision-making [35].

The tools psychometric properties were explored. The methods and result of this analysis is reported elsewhere [26].

Regression models were used, including organization (LG) as a random effect to account for clustering, to test for a linear relationship between domain scores and key variables (e.g. culture and budget). Random effects regression models were used as the analysis was interested in the variance across both organizations and individuals.

To ensure immersion in the qualitative data, each interview was reviewed three times (RA). Interviewees were also sent their transcripts for review, and any errors in the transcripts were amended. Qualitative data were then imported into spreadsheets for coding, sorting and organizing [36]. Open coding and constant comparative method [37] were used to identify emerging themes and to explore the relationships between themes [36]. Codes were generated for each of the three elements of EIDM. Emerging issues were considered and noted during data collection, which also helped to inform and strengthen interviews as they progressed. Given that knowledge translation perspectives and theory were broadly guiding the overall study processes, a grounded theory constructivist approach to data collection and analysis was deemed not entirely applicable. Reflexivity in this research was addressed by multiple researchers in the team having input to the formulation of the questions, data collection and analyses. RA conferred with investigators and kept field notes in part to identify and acknowledge researcher impact on the research process.

### Survey participation

Forty-five LGs agreed to survey participation (overall participation rate = 57%). The sample included similar number of rural (*n* = 22, 49%) and metropolitan (*n* = 23, 51%) LGs, representing most metropolitan LGs in the state (22 of a total possible 31 = 71%) and nearly half of all regional LGs (23 of a total possible 48 = 48%). As expected, sample characteristics such as budget, population size and geographic region size varied considerably (Table 1).

**Table 1:** **Characteristics of participating and non-participating councils**

	**Population size**	**Recurrent income (AUD$ million)**	**Geographic size (km** ^**2**^ **)**	**Metro/rural**
Participating councils	Mean: 77,106	Mean: 84	Mean: 2,425	M: 22
	SD: 56,330	SD: 69	SD: 593	R: 23
Non-participating councils	Mean: 49,577	Mean: 52	Mean: 3,487	M: 9
	SD: 55,877	SD: 42	SD: 3,903	R: 25

*SD* standard deviation, *M* metro, *R* rural.

From a possible 180 respondents, 135 completed the statewide survey (75% response rate, estimated based on the offer of four invitations per LG). Characteristics of individual respondents from all 45 participating LGs are shown in Table 2.

**Table 2:** **Characteristics of individual respondents**

**Baseline characteristics**	**Responses (** ***n*** **)**
Gender (*n* = 135)	
Female	85 (63%)
Male	49 (37%)
Age group (*n* = 134)	
18–19	0 (0%)
20–29	20 (14.9%)
30–39	33 (24.6%)
40–49	40 (29.9%)
50–59	37 (27.6%)
60+	4 (3.0%)
Years in LG (*n* = 134)	
Mean	10.53
Median	8
Range	0.8–33.0
Years in current position (*n* = 135)	
Mean	3.85
Median	2.5
Range	0.06–30.0
Highest level of qualifications (*n* = 135)	
Primary school	0 (0%)
Secondary school	4 (3.0%)
Certificate	2 (1.5%)
Advanced diploma/diploma	15 (11.1%)
Bachelor degree (including honour degrees)	46 (34.1%)
Graduate diploma/graduate certificate	42 (31.1%)
Postgraduate degree (masters or PhD)	26 (19.3%)

Note: where sample size is less than *n* = 135, this indicates missing data for that item.

### Interview participation

Ninety-eight people volunteered to be interviewed and 19 people were contacted using a sampling framework built previously using maximum variation sampling techniques to identify various decision-making experiences [38]. Of these, 13 interviewees were identified and 6 were non-respondents. Interviewees were selected on the basis of their position title (e.g. environmental health or social planning), geography (rural/metro) and seniority (e.g. project officer or senior manager). Interviews continued until data saturation was reached, i.e. no new data were emerging to describe the processes of EIDM operating in LGs [36].

### Defining evidence

The EvIDenT survey did not ask participants to define evidence. Rather, it provided broad definitions as response options and focused on research evidence, which was defined for the purposes of clarity.

Interviews provided insight into how evidence was defined by both individuals and their organizations. Analysis revealed a lack of consensus amongst interviewees about what constitutes ‘evidence’. Evidence was defined across a spectrum encompassing academic research, local research and evaluation, policy documents, population level or local data, community views, collegiate expertise and professional experience. In most cases, interviewees cited a combination of sources as forming an ‘evidence base’ to inform decision-making, for example community views plus academic research and local data. There was a strong focus on ‘evidence’ as defined by population-level data including census data and burden of disease data. Academic research was considered by many respondents as a crucial form of ‘evidence’. Interestingly, many participants struggled to provide a clear definition of evidence.

Participants were asked to identify whether their own perceptions of evidence were shared by their organization. Whilst some felt that there was shared clarity around what constituted evidence across the organization, others felt the definition differed, depending on which department they worked in or on their level of seniority within the organization.

### Types of evidence used and decision-making processes

Interviews also revealed the different types of evidence used to inform the development of priorities versus the development of strategies (that is, those that more specifically guide action). Population-level data (derived from either census, burden of disease or locally conducted surveys) were commonly used to inform priority setting, either sourced at the start of a planning process, simultaneously with or after community consultation. Strategy development appeared to be a more collaborative process, including consultation and data gathering. ‘*Ultimately the consultation phase is the key, or what we are told by services or by community groups is where we focus our activities*’ [KI11].

### Access, confidence and organizational culture

**Figure 1:**
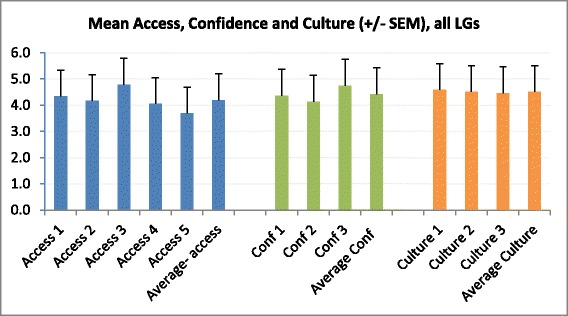
**Mean access, confidence and culture scores.** Access 1: It is easy for me to access the most relevant research findings available as I plan programs and policies. Access 2: It is easy for me to access someone who can provide help in finding, interpreting and using research findings (e.g. librarian, epidemiologist or researcher). Access 3: I have access to government reports that I need to inform decision-making. Access 4: I have access to academic literature that I need to inform decision-making. Access 5: I have access to synthesis or collations of academic literature (e.g. systematic reviews) that I need to inform decision-making. Confidence 1: How confident do you feel about your ability to find academic literature? Confidence 2: How confident are you in assessing the quality or trustworthiness of sources of evidence? Confidence 3: How confident are you in combining different sources of research evidence to inform decision-making (e.g. different journal articles and reports)? Culture 1: Overall, the culture in my local government is one that highly values the use of research evidence in decision-making for program planning. Culture 2: Research evidence is consistently included in the decision-making process related to program planning, implementation and evaluation in my local government. Culture 3: This local government is influenced by research evidence when making decisions about public health programs.

Whilst the interviews explored experiences with evidence and the processes of EIDM in LGs, the survey aimed to determine levels of access to evidence, confidence in finding and using evidence, and LG culture for EIDM (see Figure 1).

#### Access

Respondents generally reported moderate levels of access to a range of resources, whilst access to syntheses of evidence, and someone to help make sense of evidence, was lower. Interviewees discussed a lack of access to databases and therefore sources of research evidence. As a result, many relied on policy reports or evidence synthesis produced by government departments or peak bodies and Internet searches to provide findings from research evidence.

#### Confidence

Levels of confidence in searching, assessing quality and combining sources of evidence were moderate to high amongst many respondents (58.2%). Levels of reported confidence in searching for academic literature and assessing the quality or trustworthiness of sources of evidence varied. For example, whilst 26.0% reported high levels of confidence (scoring 6/7) in searching for academic literature more than a third (32.8%) had lower levels of confidence (scoring 1–3). Confidence was not discussed in the interviews.

#### Culture

Respondents generally reported a moderate to high organizational culture for supporting EIDM. However, when asked for overall ratings of organizational culture, a quarter of respondents provided low ratings (i.e. 1, 2 or 3/7) suggesting some variation in responses between LGs. The culture of EIDM was discussed extensively in the interviews. The interviews revealed that there appeared to be an expectation in some LGs, or within some teams, that evidence be used to inform decisions. For example: ‘*Some of the bigger—bigger strategies, you know, the health and well-being plan, broad service strategies …All of those things definitely have to delve into the research. You have to have it well referenced*’ [KI18]. However, this did not often appear to be written down or formally enforced. In other LGs, there was limited imperative to use research evidence: ‘*So there is no real need to produce rigorous, you know, peer reviewed programs and services …I like that in some ways. In other ways it’s a bit of a shame that there is no-one really monitoring this stuff*’ [KI8]. For some interviewees, a lack of organizational culture supporting EIDM did not appear to stop them using evidence in internal decision-making.

Whilst many of the interviewees noted increasing acknowledgement of the need to practice EIDM within LGs, there did appear to be rhetoric associated with its use. As one interviewee noted: ‘*Evidence-based stuff is certainly bandied around in the health area. Less so in other areas. But I don’t know that it’s well understood what that means. You understand the words but you don’t understand the implications… [so] the understanding is that it’s valuable and it’s needed but the link between how it actually informs the policy or the outcome, there’s a real gap*’ [KI11]. Organizational support was also linked to time and ‘*so whilst it is certainly important, it is the first thing to fall off the list of things to do*’ [KI11].

Interestingly, the culture associated with conducting and using internally generated evaluation findings to facilitate EIDM in councils appeared to be limited. Interviewees were asked more specifically about this relationship, and there was general agreement about the link between a culture of evidence and EIDM: ‘*If you understood why you have evidence, there wouldn't be a question of you practicing it*’ [KI3].

Linear regression, where LG was fit as a random effect, was also used to test for relationships between composite scores within each of the key domain areas (access, confidence and organizational culture). This analysis revealed significant linear relationships between key domain areas (see Table 3): where access was rated highly, confidence was also likely to be rated highly (*p* = <0.01); where culture was rated highly, confidence was also likely to be rated highly (*p* = <0.05); and where culture was rated highly, levels of access were also likely to be rated more highly (*p* = <0.01).

**Table 3:** **Linear relationships between composite domain scores**

**Domains**	***p*** **value**	**CI**
Access (IV) and confidence (DV)	<0.01	0.34–0.66
Confidence (DV) and culture (IV)	<0.05	0.00–0.29
Culture (IV) and access (DV)	<0.01	0.13–0.39

### EIDM skills and relationships with access, confidence and organizational culture

Survey respondents were asked to identify whether they had participated in training that helped them to make judgments about the quality of research evidence. Half of respondents (50%) had participated in training program/s; however, many had not (41%) or were unsure (9%). Those who had undertaken critical appraisal training had a higher mean confidence summary score compared to those who had not. Regression analysis revealed a statistically significant linear relationship between skills and standardized scores for confidence (*p* = <0.05, confidence interval (CI) = 1.023, −.631) and access (*p* = <0.05, CI = .527, −.081). There was however no statistically significant relationship between skills and standardized organizational culture scores (*p* = 0.426 CI = .389, 0.164).

Skills (or a lack thereof) and skills development were a core theme of the interviews. Limited opportunities for professional development around EIDM for council staff and executive were apparent. Only one interviewee described having undertaken any relevant training. To address this lack of skill, consultants were often used to develop LG plans. A need for further professional development and accreditation or standard of skills was discussed.

### Usefulness and influence

Survey respondents were asked to rate the influence and usefulness of a range of people/groups and resources in informing public health decision-making.

People or groups with the *greatest influence* were councilors, CEO, public health managers (i.e. managers of public health departments within LGs) and the community. Academics were rated as having the least influence. Public health managers were rated the *most useful* people/groups in public health decision-making, and personal experience and the community were also highly rated. Least useful were councilors and advocacy/lobby groups. For nearly all people/groups rated, there were statistically significant linear relationships between the way influence and usefulness was rated. The exception was for councilors, who appeared to be more influential than useful in decision-making.

LG policy, plans and by-laws were the most *influential* resource in public health decision-making. Government reports were also highly rated. Similarly, the most *useful* resources were government reports, LG policy by-laws, and non-government reports. The least *influential* resources were academic reports and journal articles, and general published literature. The least *useful* resources were general published literature and newsletters/bulletins or online alerts.

To summarize views on ‘influence’ and ‘usefulness’, respondents were asked to identify the types of evidence that have the greatest influence or those of most use in decisions. A ‘mixture of evidence’ was commonly reported as being *most influential* on public health planning decisions (93.9%). Of these, 55.7% favoured a mixture of evidence with ‘*more internal* than external’ evidence. There was no impact of clustering (measured by intracluster correlation coefficient (ICC)) upon the variability in the views of respondents within LGs (ICC = 0.00). That is, individuals within LGs were differentially influenced by internal, external, or a mix of influences.

Similarly, a mixture of evidence was commonly reported as being *most useful* for informing public health planning decisions (94.6%). Of these, 56.1% favoured a mixture of evidence with ‘*more external* than internal’ evidence. Again, there was no impact of clustering on the identified variability in the views of respondents within the same LG (ICC = 0.00). The individual variation in responses to this question within LGs suggests that individuals may find evidence more or less useful than others within the organization.

The interviews also revealed a number of influences on decision-making, which were categorized as direct and indirect influences (see Additional file 2). Direct influences were those that had a direct impact on individual public health decisions, and indirect influences were those that had a more distal influence. Discussions of the usefulness of evidence sources were only briefly described. As described above, community consultations were highly valued in the decision-making process.

### Barriers and facilitators to using evidence to inform decision-making

Barriers and facilitators, together with their relationship to core EIDM domains, are summarized in Additional file 3. Rated as the highest barrier was ‘time to look for evidence’ (mean score 4.9/7) and the lowest-rated was ‘uncertainty of the evidence base’ (mean score 3.7/7). Whilst ‘confidence in using research evidence’ was also highly rated as a barrier (mean score 4.8/7), respondents also rated the issue of ‘further development of skills in finding, accessing and using evidence’ very highly (mean score 4.9/7).

Linear regression was used to further explore relationships between the core domains (independent variable) and barriers and facilitators (dependent variable) to EIDM. A number of significant relationships were identified (see Additional file 4).

Consistent with the survey findings, the skill of staff was cited as both a facilitator and barrier to EIDM in the interviews. Skilled staff or program champions were acknowledged as important facilitators of an EIDM approach**.** Lack of research and evaluation skills were acknowledged as a barrier. As a result, there were challenges for staff in identifying ‘*what are the key issues, how we’re going to measure them, you know, where we’re going to start getting the information from, and how we’re going to report on it*’ [KI2]. Many therefore relied on policy reports or evidence synthesis produced by government departments or peak bodies and on Internet searches, to provide findings from research evidence.

Whilst some interviewees identified that having time to read and make sense of research evidence would assist the EIDM process, time to do this was limited. Time emerged as a connecting influence; that is, it was linked to all other direct influences including skill, access, organizational support and presentation of the evidence.

### Council budget as a determinant of EIDM practice and culture

It was anticipated that LG budget would be linked to the resources available to practice EIDM. To help to confirm the importance of budget, population size within LGs was plotted against their budgets, revealing a linear association. LGs with lower budgets were more likely to serve smaller populations. Similar graphs were drawn between other key variables but these appeared to be less linear. Based on this analysis, it was proposed that budget or population size served could plausibly be selected as key variables for randomisation in the proceeding intervention. Given the resource implications of practicing EIDM, budget was deemed to be the most appropriate variable.

## Discussion

EIDM is increasingly promoted in public health [5]. Its importance is lauded from an effectiveness, cost-effectiveness and ethical perspective [3,39-41]. However, this study argues that there are challenges associated with an evidence-informed approach: the availability of research evidence, the type of research evidence available and the inadequacy of research evidence [3,42,43]. In particular, it provides a unique perspective of these issues for LG agencies, which are inherently multi-sectoral and where evidence must be drawn from various sources to inform local decisions.

As theoretical perspectives suggest, decision-making is inherently political and even where research evidence is available, it needs to be tempered with a range of other sources of evidence including community views, financial constraints and policy priorities [4-6,44]. This aligns with our participants’ perspectives on evidence as representing a wide range of sources and resources. Definitions of evidence included academic research, local research and evaluation, policy documents, population-level or local data, community views, collegiate expertise and professional experience. These can be referred to as type 1 (evidence to describe problems for priority setting) and type 2 evidence (evidence of effectiveness to aid strategy development) [42,43,45]. However, there appeared to be a strong preference for data (type 1 evidence). Given this, it is perhaps not surprising that this study revealed that evidence was often applied more commonly to priority setting process than strategy development [46]. This may limit effectiveness and cost-effectiveness and may cause harm [39,40,47]. It was hard to deduce whether evidence to support implementation (type 3) was used, although interviewees did not specifically mention this type of evidence. This highlights a potential point for knowledge translation interventions to address and the need for better links between researchers and decision-makers.

Analysis revealed varying levels of perceived access, confidence and organizational culture to support EIDM. These domains, informed by theoretical frameworks, were developed as determinants of EIDM. To date, little evidence is available to allow comparisons of this finding. This study’s comparisons between EvIDenT findings and interview data present some opportunity to explore these concepts. The interviews revealed that access to electronic databases was a perceived barrier. This is likely linked to the fact that LG staff rarely have full-text access to electronic databases. However, many online resources are available online for free (e.g. Cochrane Library and health-evidence.org) and so increasing awareness of these resources may alter these perceived levels of access. Survey results revealed strong correlations between access, confidence and organizational culture. This suggests that interventions to support EIDM may be strongest when each of these elements is collectively addressed. Given that a lack of training was a barrier to EIDM, workforce development should be considered for LG staff particularly those in management, which may help create a stronger culture for EIDM within teams. Activities that promote meaningful exchange between researchers and decision-makers may also assist in expanding EIDM culture within organizations [23,48,49].

Culture emerged as an important issue in supporting EIDM. This is an ongoing challenge for organizations where clear processes are not in place to guide staff on how to source, appraise and combine different sources of evidence to decide on interventions [50]. This lack of organizational leadership also emerged in this study. Although we acknowledge that one set process is unlikely to work for multiple organizations, we sought to understand whether organizational processes existed to support individuals to practice EIDM in their context. In this sample, there were limited organizational processes for evidence-informed strategy development, although some reflected on the need to be responsive rather than strategic when making decisions about public health actions. In addition, some interviewees were unable to identify whether their own perceptions of evidence were even shared by their organization, again suggesting a lack of organizational culture and leadership.

This paper presents an emerging picture of decision-making within LG. The EvIDenT survey identified the degree to which different forms of evidence are useful and influential. Influence is well described in the literature and can emanate from both internal and external sources [18,20,51]. We chose to differentiate between influence and usefulness as some sources of evidence may be influential in decision-making but not deemed to be useful (e.g. appropriateness/relevance or vice versa). Resources found to be both influential *and* useful included council policy, plans and by-laws and government reports; whilst academic reports, journal articles and general published literature were reported to be the *least* influential when making decisions about decision-making within their LG. Public health managers and the community were identified as both useful and influential.

We also chose to differentiate between internal and external evidence, defining internal evidence for participants as organizationally derived evidence, including organizational data and community opinions. External evidence was defined as peer-reviewed research or policy frameworks from other contexts. Overall, a mixture of evidence, but more *internal* than external evidence, was *influential* in public health decision-making in LGs. By comparison, a mixture of evidence, but more *external* than internal evidence, was deemed to be *useful* in public health decision-making. This suggests that internal evidence, which may not be tested for rigour, may be more influential in LGs. Participants recognized the usefulness or importance of external evidence in guiding decision-making. Research using these concepts is not available in comparable populations [24]. Interviews confirmed these results but more specifically identified the diversity in EIDM application processes across LGs. The influence of external evidence has been documented [5], but the interaction between use and influence is less well understood. This study also revealed differences between usefulness and influence ratings for CEOs. That is, CEOs were deemed to be more influential than useful, a power implication which is important in understanding how evidence is used [52]. This link between the importance of organizational support and a culture of EIDM highlighted in the interviews and usefulness and influence requires more investigation.

The barriers to EIDM have been well documented and include time, access to resources, organizational culture, political influences, and skill in finding and using research evidence [1,19,53,54]. As identified in previous research [54], time was one of the most dominant barriers to EIDM in this study. Whilst the need for skills development to support EIDM practice was highly rated, so was confidence in using the evidence. Further research is needed to explore this difference in perceptions. This study sheds some light on the skills of those working in LGs to practice EIDM, and whilst it was not identified as a core domain, it emerged as an important factor. Many interviewees discussed the skill set of LG staff as either a facilitator or barrier to EIDM. Professional background also emerged as an important factor, given that many identified as coming from a diverse range of professional backgrounds. This is supported by survey results, which identified that only 41% of participants had undertaken critical appraisal training. It may be useful to extend the EvIDenT survey to explore skills in accessing and applying evidence, in addition to a focus on evidence assessment [55]. A stronger focus on organizational capacity is also needed [56].

This study benefited from the use of mixed methods design and analysis. The concurrent studies presented a detailed overview of the decision-making processes undertaken in LG. The survey revealed new data about access to evidence, confidence in using evidence and organizational culture for EIDM in LG. The interviews helped to explore the influences on these domains.

### Limitations

This study involved 135 participants drawn from 45 LGs (more than half of all LGs in the state of Victoria). Even so, this presents issues for broader generalisability of these findings. Those who completed the EvIDenT survey may have had more interest in EIDM processes, which could account for moderate to high levels seen in scores. Despite the small sample, the commonalities identified in responses across LGs indicate that those outside of the study sample may share many of the issues described in this study. Further research with a larger sample may provide a more complete picture of how EIDM operates [57]. Given that the influence of councilors and the community is seen, it would be beneficial to include these populations in subsequent research. Previous research has identified the need to consider organizational structural features, culture and beliefs, leadership style and resources as barriers to evidence-informed public health decision-making [58]. Further research may be needed to more adequately capture the culture of EIDM in public health agencies including LGs.

Whilst the EvIDenT survey was not tested for reliability, it was extensively piloted. Given the complexity of decision-making in policy contexts, the use of survey methodology presented some challenges in terms of gaining a complete picture of current activity. This was resolved by incorporating a qualitative component.

## Conclusions

The findings from this study describe how evidence is defined and used in a multi-sectoral LG setting. Government policy has articulated the need for evidence to inform local policy and planning, and the importance of EIDM in public health is acknowledged as important to improve population health. However, the results demonstrate that there is much to be done to build organizational culture to support EIDM practice.

The EvIDenT survey is one of few tools developed to enable exploration of EIDM in a community-based public health setting. It was designed to help explore how evidence is used within LG; to summarize the usefulness and influence of a range of sources of evidence and provide insight into how research is accessed, the level of confidence associated with research use, and the extent of an underlying organizational culture of EIDM. Given the breadth of the questions, it is likely to have broader application beyond LG.

The findings presented in this paper provide a unique picture of how LGs make public health decisions. The results highlight the influence of some forms of evidence (e.g. community views) at the expense of others (e.g. research evidence). This suggests the need for enhanced organizational and system-level support to improve levels of access and confidence in using research evidence. Increased transparency requirements may encourage the consideration of various sources of evidence. Stronger organizational culture may result from such measures but may require more targeted interventions at either a state or regional level.

Redressing the challenges to the use of evidence in LG decision-making identified in this paper is complex. The decision-making process will always be political and the time pressures for staff will always be significant. Building a stronger normative culture for EIDM is needed to ensure that decisions relevant to population health outcomes are adequately informed by research evidence, an expectation that would be considered standard in any other contexts where health outcomes are affected.

## Additional files

Additional file 1:
**Baseline survey items mapped to source and domain.**
Additional file 2:
**Influences on local government public health decision-making.**
Additional file 3:
**Extent of agreement (or disagreement) between barriers and facilitators to EIDM.**
Additional file 4:
**Relationships between barriers and facilitators and core domains of EIDM: linear regression significance and confidence intervals.**

## Abbreviations

EIDM: evidence-informed decision-making
KT4LG: Knowledge Translation for Local Government (study title)
LG: local government
