# Supplementary material for: Understanding evidence: a statewide survey to explore evidence-informed public health decision-making in a local government setting
Source: Implement Sci. 2014 Dec 14;9:188. doi: 10.1186/s13012-014-0188-7 (PMC4314798; doi:10.1186/s13012-014-0188-7)
Supplement: Additional file 1: — Baseline survey items mapped to source and domain. [file 13012_2014_188_MOESM1_ESM.docx]

Additional file 1. Baseline survey items mapped to source and domain

| **Item #** | **Item** | **Source** | **Changes from standard** | **Related domain** |
| --- | --- | --- | --- | --- |
| 1 | Are you male or female |  |  | Demographic |
| 2 | What is your age group? | Australian Bureau of Statistics standard |  | Demographic |
| 3 | What is the post-code of your work address? |  |  | Demographic |
| 4 | What is your position within your organisation? | Informed by pre-pilot, Dobbins et al [26]. | What is your current position at the organization (Q1] | Demographic |
| 5 | How many years have you worked within local government? | Dobbins et al.[26] | Asked about years of experience in public health but we have changed the emphasis to years in LG [Q3] | Demographic |
| 6 | In which area or department of your local government do you currently work? | NEW - to capture LG context |  | Demographic |
| 7 | How many years of experience do you have in your current position? | Dobbins et al.[26] |  | Demographic |
| 8 | What is your highest level of educational qualifications? | Australian Bureau of Statistics standard |  | Demographic |
| 9 | If you have post-secondary school qualifications, what is the professional training that you align most closely? | Australian Bureau of Statistics standard, Dobbins et al. [26] | What is your professional background in terms of the subject matter of your initial university degree [Q4] Australian Bureau of Statistics = broadened standard of 'health' to include areas of health | Demographic |
| 10 | In what way were you involved in the development of your local governments most recently published Municipal Public Health Plan? | NEW - informed by pre-pilot |  | Demographic |
| 11 | It is easy for me to access the most relevant research findings available as I plan programs or policies | Dobbins et al.[26] | It is easy for you to access the most relevant research available as you plan programs or policies [Q24] | Access |
| 12 | It is easy access to someone who can provide help in finding, interpreting and using research findings (e.g. librarian, epidemiologist or researcher) | Dobbins et al.[26] | I have easy access to someone who can provide help in interpreting or utilising research evidence [this person could be a librarian, epidemiologist, or academic - anyone who can help you to understand research] [Q19] | Access |
| 13 | I have access to government reports that I need to inform decision-making | NEW - The following three items (13-15) were added to assess level of access to a range of sources of evidence. Although having access and knowing where to access are two different things. If these items are about knowledge then perhaps that needs to be the focus. Pre-tested items for knowledge and confidence were not identified. Used existing surveys [25, 26] to help construct these items |  |  |
| 14 | I have good access to academic literature that I need to inform decision-making? | NEW - see item 13 |  | Access |
| 15 | I have good access to synthesis or collations of academic literature e.g. systematic reviews that I need to inform decision-making | NEW - see item 13 |  | Access |
| 16 | How confident do you feel about your ability to find academic literature? | NEW – designed to collect data on confidence |  | Confidence |
| 17 | How confident are you in assessing the quality or trustworthiness of sources of evidence? | NEW - designed to collect data on confidence |  | Confidence |
| 18 | Have you ever participated in training program/s that helped you to understand how to make judgements about the quality of research evidence? This content is sometimes referred to as critical appraisal [plus definition]. | NEW – included to identify competency level |  | Skills |
| 19 | How confident are you in combining different sources of research evidence to inform decision-making e.g. different journal articles and reports? | NEW – based on diffusion of innovations theory [19] and informed by Weatherly et al. [25] | What kind of evidence do you think would help to improve the decision-making process when faced with a choice between different interventions for either Coronary Heart Disease or Cancer? [Q21] | Confidence |
| 20 | Overall, the culture in my LG is one that highly values the use of research evidence in decision-making for program planning | Dobbins et al. [26] | Overall, the culture in my organization is one that highly values the use of research evidence in decision-making for program planning [Q16] | Organisational culture |
| 21 | Research evidence is consistently included in the decision-making process related to program planning, implementation and evaluation in my LG | Dobbins et al.[26] | Research evidence is consistently included in the decision-making process related to program planning, in my organization. [“included” means that it is one piece of information that is used in the decision-making process] [Q18] | Organisational culture |
| 22 | This local government is influenced by research evidence when making decisions about public health programs | Dobbins et al.[26] | The governing board of my organization is influenced by research evidence when making decisions about public health programs. [“research evidence” refers to scientific studies] [Q21] | Organisational culture |
| 23 | The following people and groups may INFLUENCE public health decision making. Please rate each item on the scale provided from not influential to very influential, as relevant to your local government. | Weatherly et al.[25] | For the HImP program that you have chosen to focus on, how important were the following sources of internal evidence in deciding what Coronary Heart Disease/Cancer services to provide to meet priorities identified [Q10] | Influence |
| 24 | The following resources may INFLUENCE public health decision making. Please rate each item on the scale provided from not influential to very influential, as relevant to your local government. | Weatherly et al.[25] and informed by the pre-pilot. | For the HImP program that you have chosen to focus on, how important were the following sources of internal evidence in deciding what Coronary Heart Disease/Cancer services to provide [Q11] |  |
| 25 | The following people and groups may be USEFUL in public health decision making. Please rate each item on the scale provided from not useful to very useful, as relevant to your local government. |  |  | Usefulness |
| 26 | The following resources may be USEFUL in public health decision making. Please rate each item on the scale provided from not useful to very useful, as relevant to your local government. | Weatherly et al.[25] and informed by the pre-pilot. |  | Usefulness |
| 27 | Overall, which source of information has the greater INFLUENCE on public health planning decisions in your local government? | Weatherly et al. [25] | Generally speaking, we are interested to know about the type of evidence used to decide which health care interventions to focus on (for either Coronary Heart Disease or Cancer). Would you say the evidence was internal evidence OR external evidence?[Q11] | Influence |
| 28 | Overall, which source of information is most USEFUL for public health planning decisions in your local government? | Weatherly et al. [25] | Generally speaking, we are interested to know about the type of evidence used to decide which health care interventions to focus on (for either Coronary Heart Disease or Cancer). Would you say the evidence was internal evidence OR external evidence?[Q11] | Usefulness |
| 29 | The following people and groups, as outlined previously, may be involved in public health decision making related to obesity prevention in children. Focusing on decisions related to programs or activities for obesity prevention in children, please rate each item’s USEFULNESS on the scale provided, from not useful to very useful, as relevant to your local government. | Weatherly et al.[25] |  | Usefulness |
| 30 | The following resources, as outlined previously, may be involved in public health decision making related to obesity prevention in children. Focusing on decisions related to programs or activities for obesity prevention in children, please rate each item’s USEFULNESS on the scale provided, from not useful to very useful, as relevant to your local government. | Weatherly et al. [25] |  | Usefulness |
| 31-40 | What do you think are the main barriers and enablers to using research evidence in public health decision-making? | Weatherly et al.[25] | What do you think are the main barriers to using evidence in HImP design are? [Q20] | Barriers and facilitators |
| 41 | Describe one of the innovative ways your organisation shares knowledge about effective programs | NEW – Informed by diffusion of innovations theory [19] |  | Describing current practice and informing intervention design |
| 42 | What additional strategies do you think would be useful to share evidence? | NEW – Informed by diffusion of innovations theory [19] |  | Describing current practice and informing intervention design |
| 43 | Overall, how do you feel you could be better supported to make use of evidence in your role? | NEW – Included to inform intervention design |  | Describing current practice and informing intervention design |
