# Supplementary material for: Understanding evidence: a statewide survey to explore evidence-informed public health decision-making in a local government setting
Source: Implement Sci. 2014 Dec 14;9:188. doi: 10.1186/s13012-014-0188-7 (PMC4314798; doi:10.1186/s13012-014-0188-7)
Supplement: Additional file 2: — Influences on local government public health decision-making. [file 13012_2014_188_MOESM2_ESM.docx]

**Additional File 3. Influences on local government public health decision-making**

| **Direct influences** | **Indirect influences** |
| --- | --- |
| Skill of staff | Community |
| Access to evidence | Councillors |
| Presentation of evidence | Council size and structure |
| Organisational support | Statutory focus |
| Time | Local connections |
