# Supplementary material for: Understanding evidence: a statewide survey to explore evidence-informed public health decision-making in a local government setting
Source: Implement Sci. 2014 Dec 14;9:188. doi: 10.1186/s13012-014-0188-7 (PMC4314798; doi:10.1186/s13012-014-0188-7)
Supplement: Additional file 3: — Extent of agreement (or disagreement) between barriers and facilitators to EIDM. [file 13012_2014_188_MOESM3_ESM.docx]

Additional file 4. Extent of agreement (or disagreement) between barriers and facilitators to EIDM

|  | **% and mean total of respondents rating barriers and facilitators as high or low** | | |
| --- | --- | --- | --- |
|  | **Rating 1 or 2 (%)**  **(strongly disagree/disagree)** | **Rating of 6 or 7 (%)**  **(agree/strongly agree)** | **Mean rating (range = 1 strongly disagree -7 strongly agree)** |
| Relevant evidence is available (n=131) | 4.6 | 29.8 | 4.7 |
| Relevant evidence is sufficiently accessible (n=131) | 15.3 | 22.9 | 4.2 |
| The evidence is understandable (n=130) | 20.8 | 23.9 | 4.6 |
| The evidence is too uncertain to adequately inform decision-making (n=131) | 16.0 | 8.4 | 3.7 |
| There is not enough time to look for evidence (n=131) | 6.9 | 36.6 | **4.9** |
| There is too much information to work with (n=129) | 10.9 | 24.8 | 4.3 |
| There is not enough time to fully understand the evidence findings for my context (n=130) | 10.8 | 33.1 | 4.5 |
| I prioritise my time to find and use evidence (n=130) | 8.5 | 24.6 | 4.4 |
| I’d like to develop my skills further in finding, accessing and using evidence (n=130) | 10.0 | 41.5 | **4.9** |
| Overall, I feel confident enough to use evidence (n=131) | 3.8 | 32.1 | **4.8** |
