# Supplementary material for: Understanding evidence: a statewide survey to explore evidence-informed public health decision-making in a local government setting
Source: Implement Sci. 2014 Dec 14;9:188. doi: 10.1186/s13012-014-0188-7 (PMC4314798; doi:10.1186/s13012-014-0188-7)
Supplement: Additional file 4: — Relationships between barriers and facilitators and core domains of EIDM: linear regression significance and confidence intervals. [file 13012_2014_188_MOESM4_ESM.docx]

Additional File 5. Relationships between barriers and facilitators and core domains of EIDM: linear regression significance and confidence intervals

| **Barriers and facilitators** | **Access** | **Confidence** | **Culture** |
| --- | --- | --- | --- |
| Relevant evidence is available | **p=<0.01**  **CI=0.46,0.97** | **p=<0.01**  **CI=0.28,0.76** | **p=<0.05**  **CI=0.01, 0.45** |
| Relevant evidence is sufficiently accessible | **p=<0.01**  **CI=0.51,1.08** | p=0.19  CI=-0.10,0.49 | **p=<0.01**  **CI=0.17,0.66** |
| The evidence is understandable | **p=<0.01**  **CI=0.44,0.93** | **p=<0.01**  **CI=0.15,0.63** | p=0.05  CI=0.00,0.41 |
| The evidence is too uncertain to adequately inform decision-making | p=0.39  CI=-0.15,0.40 | p=0.25  CI=-0.4,0.11 | p=0.06  CI=-0.01,0.42 |
| There is not enough time to look for evidence | p=0.28  CI=-0.49,0.14 | p=0.83  CI=-0.26,0.33 | p=-0.63  CI=-0.31,0.19 |
| There is too much information to work with | p=0.16  CI=-0.54-0.09 | p=0.30  CI=-0.45,0.14 | p=0.17  CI=-0.07,0.43 |
| There is not enough time to fully understand the evidence findings for my context | **p=<0.05**  **CI=-0.64,-0.03** | **p=<0.05**  **CI=-0.58,-0.02** | p=0.34  CI=-0.13,0.37 |
| I prioritise my time to find and use evidence | **p=<0.05**  **CI=0.18,0.74** | **p=<0.01**  **CI=0.42,0.91** | **p=<0.05**  **CI=-0.05,0.46** |
| I’d like to develop my skills further in finding, accessing and using evidence | p=0.83  CI=-0.36,0.29 | **p=<0.05**  **CI=-0.66,-0.07** | p=0.11  CI=-0.05,0.46 |
| Overall, I feel confidence enough to use evidence | **p=<0.05**  **CI=0.06,0.57** | **p=<0.01**  **CI=0.63,1.03** | p=0.28  CI=-0.09,0.32 |
